# Effects of Target Size and Test Distance on Stereoacuity

**DOI:** 10.1155/2016/7950690

**Published:** 2016-08-21

**Authors:** Yo Iwata, Fusako Fujimura, Tomoya Handa, Nobuyuki Shoji, Hitoshi Ishikawa

**Affiliations:** ^1^Doctor's Program of Medical Science, Kitasato University Graduate School, Kitasato, Sagamihara 252-0373, Japan; ^2^Department of Rehabilitation, Orthoptics and Visual Science Course, School of Allied Health Sciences, Kitasato University, Kitasato, Sagamihara 252-0373, Japan; ^3^Department of Ophthalmology, School of Medicine, Kitasato University, Kitasato, Sagamihara 252-0373, Japan

## Abstract

Target size and test distance effects on stereoacuity were investigated in 24 subjects using a three-dimensional monitor.* Examination 1: Target Size Effects*. The test distance was 2.5 m for 0.1°, 0.2°, 0.5°, and 0.9° target sizes; crossed parallax was presented in 22-second units. Average stereoacuity values for 0.1°, 0.2°, 0.5°, and 0.9° target sizes were 59.58 ± 14.86, 47.66 ± 13.71, 41.25 ± 15.95, and 39.41 ± 15.52 seconds, respectively. Stereoacuity was significantly worse with a 0.1° target than with 0.2°, 0.5°, and 0.9° target sizes (*P* = 0.03, *P* < 0.0001, and *P* < 0.0001, resp.).* Examination 2: Test Distance Effects*. Test distances of 2.5, 5.0, and 7.5 m were investigated for a 0.5° target size; crossed parallax was presented in 22-second units. Average stereoacuity values at 2.5 m, 5.0 m, and 7.5 m test distances were 44.91 ± 16.16, 34.83 ± 10.84, and 24.75 ± 7.27 seconds, respectively. Stereoacuity at a 7.5 m distance was significantly better than at distances of 2.5 m and 5.0 m (*P* < 0.0001 and *P* = 0.02, resp.). Stereoacuity at a 5.0 m distance was significantly better than at 2.5 m (*P* = 0.04). Stereoacuity should be estimated by both parallax and other elements, including test distance and target size.

## 1. Introduction

Stereoacuity tests can be carried out easily and quickly to detect strabismus and amblyopia and to judge the degree of binocular vision after refractive correction [1–5]. Some patients who are diagnosed as having no stereopsis by conventional stereoacuity tests, such as the Titmus stereo test and TNO stereo test, can enjoy three-dimensional (3D) movies [6]. There are many differences between stereoacuity test devices used in clinical ophthalmology and movies and attractions that use 3D technology, such as whether they are static or dynamic, the target size, and the test distance. In the past, it was reported that a dynamic stereo target was more easily recognizable than a static stereo target [6–8]. Devices also differ in test distance, target size, and binocular separation method [9–15]. Recently, not only the near stereoacuity test, but also the far stereoacuity test has been used widely in clinical ophthalmology [16–18]. There are many reports demonstrating that the far stereoacuity test is superior for detection of an abnormality (especially intermittent exotropia) versus the near stereoacuity test [17, 19–24]. In addition, there is a report that stereoacuity, which the near stereoacuity test cannot detect, is detected by the far stereoacuity test [15]. To date, many studies on the effect of test distance on stereoacuity have been performed. However, in previous studies, there are various opinions about the effect of test distance on stereoacuity as a far stereoacuity test was easier to recognize than near one [25], there was no difference between far and near stereoacuity test [26–30], and it depended on the subjects [31–33]. However, the binocular separation method was not consistent, and target size did not necessarily correlate with test distance in previous studies. To investigate test distance, it is necessary to consider the target size, as target size decreases as the test distance increases. No previous study has considered both target size and test distance. We investigated the effects of target size and test distance on stereoacuity using a 3D monitor that can display targets under various conditions, and we achieved our purpose.

## 2. Methods

### 2.1. Subjects

Twenty-four subjects (mean age ± standard deviation, 21.8 ± 0.8 years) participated in the study. No subject had ophthalmic disease other than minor refractive error, and each eye had distance and near vision values of −0.08 (logMAR) under full refractive correction. Far and near eye position of all subjects were less than 10 Δ. If the subjects felt fatigued during the procedure, the experiment was stopped immediately. This research conformed to the tenets of the Declaration of Helsinki and was approved by the Kitasato University Human Sciences Ethics Committee (2010-020). The methods were carried out in accordance with approved guidelines. Potential subjects gave written consent after being given detailed information about the study and their role as a participant. Informed consent was obtained from all subjects after an explanation of the nature and possible consequences of the study.

**Figure 1:**
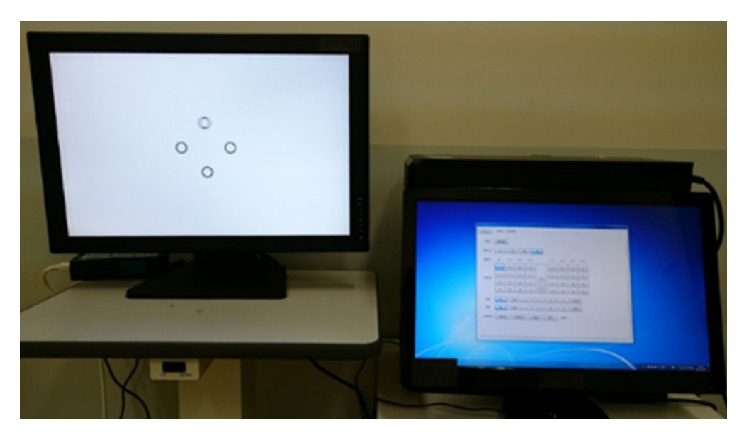
The 3D monitor (3D visual function trainer ORTe) showing the overall appearance and targets.

We carried out stereoacuity tests on the subjects using the 3D visual function trainer ORTe (Japan Focus Company, Japan). The size of 3D visual function trainer ORTe is 24 inches (518.4 (width) × 324 (height) mm) with a resolution of 1920 × 1200 pixels. We developed original software programs to display stereo targets. A polarization method (circular) was used for binocular separation. This equipment was also used for both eyes in an open visual acuity test; crosstalk is prevented, and the subject does not perceive the leakage of images (monocular cues are excluded) [34]. A 3D monitor showing the overall appearance and targets is shown in Figure 1. Analysis of variance (ANOVA) and Scheffé's method were used for statistical analysis; *P* < 0.05 was considered statistically significant.

### 2.2. Examination 1: Effect of Target Size on Stereoacuity

For this examination, the test distance was 2.5 m and the target sizes were 0.1°, 0.2°, 0.5°, and 0.9°. The target shape was a circle, and its color was black. The thicknesses of the outlines of the circles at 0.1°, 0.2°, 0.5°, and 0.9° were 1 mm, 2 mm, 4 mm, and 8 mm, respectively; the inner gap sizes were 3 mm, 6 mm, 12 mm, and 24 mm, respectively. The distance between the targets was 6 cm. The color of the background was white. The contrast between the background and target was 90%. The room illuminance was 320 lx, and the luminance of the display was 400 cd/m^2^. The amount of parallax presented using a 3D monitor depends on the distance between each pixel. Thus, parallax was presented as crossed parallax in units of 22 seconds. The presentation of parallax is limited by the resolution of the 3D monitor. Therefore, 22 seconds was the minimum parallax that could be presented at a test distance of 2.5 m. We asked the subjects to choose which of the four targets was the stereo target. The subjects answered orally. We asked the subjects to answer starting from the greatest amount of parallax (198 s) in descending order (an answer was judged correct if it was correctly answered all three times); when they responded with an incorrect answer, we judged the prior parallax as the stereoacuity.

### 2.3. Examination 2: Effect of Test Distance on Stereoacuity

The test distances for this examination were 2.5, 5.0, and 7.5 m. We designed the experiment so that the target size doubles or triples when the test distance doubles or triples. Therefore, the target sizes were 22, 44, and 66 mm; the retinal target size was met at all test distances. The target for the visual angle at all test distance was 0.5°. The thicknesses of the outlines of the circles at 2.5 m, 5.0 m, and 7.5 m were 4.4 mm, 8.8 mm, and 13.2 mm, respectively; inner gap sizes were 13.2 mm, 26.4 mm, and 39.6 mm, respectively. The distances between the targets were 5.5 cm, 11.0 cm, and 16.5 cm. The color of the background was white. The contrast between the background and target was 90%. Room luminance was 320 lx, and the luminance of the display was 400 cd/m^2^. Parallax was presented as crossed parallax in units of 22 seconds at all distances. We asked the subjects to choose which of the four targets was the stereo target. The subjects answered orally. We asked the subjects to answer starting from the greatest amount of parallax (198 s) in descending order (an answer was judged correct if it was correctly answered all three times); when they responded with an incorrect answer, we judged the prior parallax as the stereoacuity.

## 3. Results

### 3.1. Examination 1: Effect of Target Size on Stereoacuity

**Figure 2:**
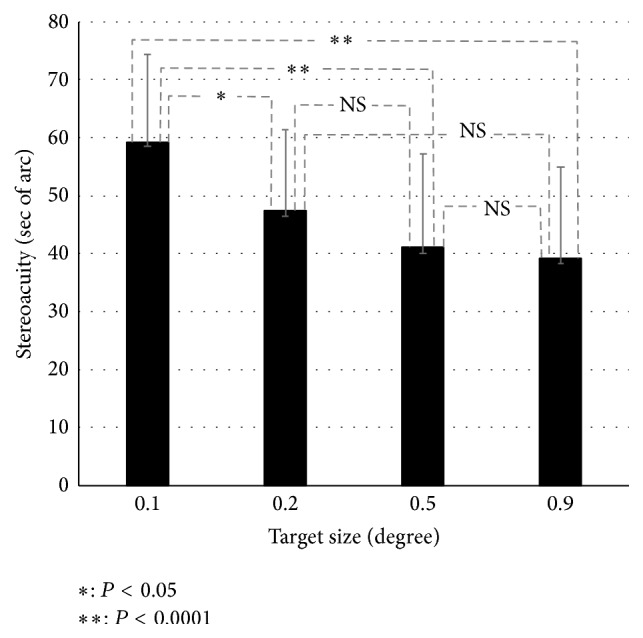
Average stereoacuity values for each target size. From the left of the graph, target sizes of 0.1°, 0.2°, 0.5°, and 0.9° are shown.

The average stereoacuity for each target size is shown in Figure 2. The average stereoacuity values at target sizes of 0.1°, 0.2°, 0.5°, and 0.9° were 59.58 ± 14.86, 47.66 ± 13.71, 41.25 ± 15.95, and 39.41 ± 15.52 seconds, respectively. ANOVA was used in order to analyze the effect of the target size in stereoacuity. The effect of the target size was significant *F*(3,69) = 21.246, *P* < 0.0001. Stereoacuity at a target size of 0.1° was significantly worse than those at target sizes of 0.2°, 0.5°, and 0.9° (*P* = 0.03, *P* < 0.0001, and *P* < 0.0001, resp.). When the target size was 0.1°, the stereoacuity decreased.

### 3.2. Examination 2: Effect of Test Distance on Stereoacuity

**Figure 3:**
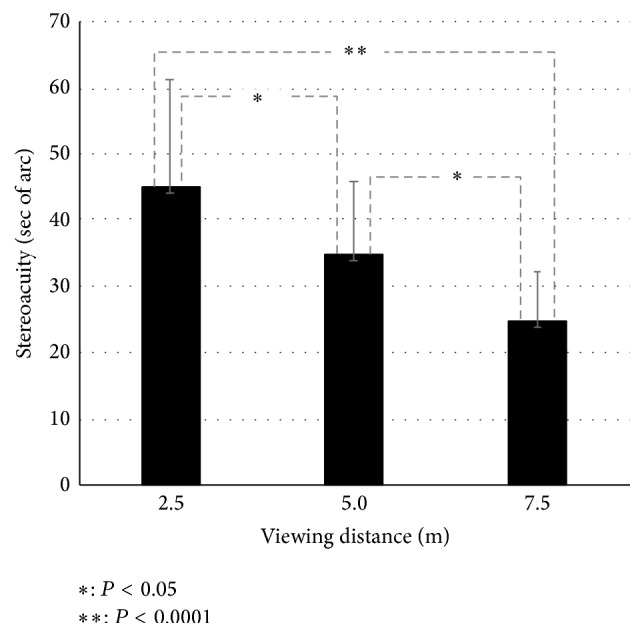
Average stereoacuity values for each target size. From the left of the graph, target sizes of 0.1°, 0.2°, 0.5°, and 0.9° are shown.

The average stereoacuity at each test distance is shown in Figure 3. Average stereoacuity values at test distances of 2.5, 5.0, and 7.5 m were 44.91 ± 16.16, 34.83 ± 10.84, and 24.75 ± 7.27 seconds, respectively. ANOVA was used in order to analyze the effect of the test distance in stereoacuity. The effect of the test distance was significant *F*(2,46) = 29.295, *P* < 0.0001. Stereoacuity at a test distance of 7.5 m was significantly better than at test distances of 2.5 and 5.0 m (*P* < 0.0001 and *P* = 0.02, resp.). Stereoacuity at a distance of 5.0 m was significantly better than at 2.5 m (*P* = 0.04). As test distance increased, stereoacuity improved.

## 4. Discussion

**Figure 4:**
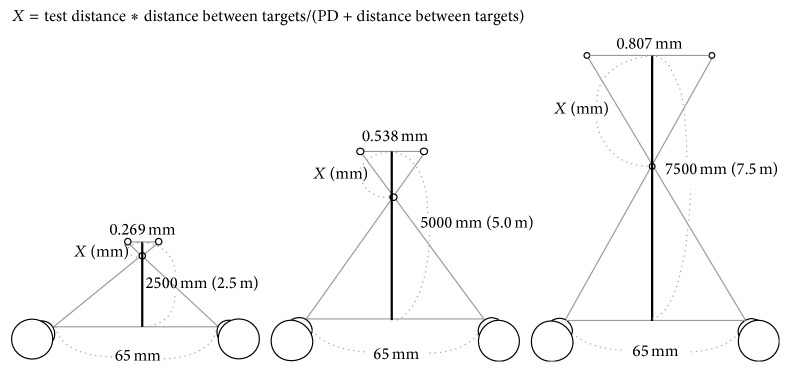
The calculation method for the projection amount.

Our results showed that stereoacuity was significantly worse when the target size was 0.1°. However, overall we observed a trend with stereoacuity becoming worse as the target size decreased. This can be explained by reduced visibility when the target size was smaller, therefore, resulting in a decrease in stereoacuity. Moreover, our results showed that stereoscopic vision improved at longer test distances. We believe that the reason in cases where the retinal target size and presented parallax were the same is that when the test distance was increased, the projection rate of the stereo target (projection amount (distance of convergence point) from the 3D monitor/test distance × 100) increased. In this experiment, for example, when the test distances were 2.5, 5.0, and 7.5 m and the presented parallax was 22 seconds, the projection amounts of the stereo targets from the 3D monitor were calculated as shown in Figure 4 for subjects with a pupillary distance of 65 mm. In the case of a 2.5 m test distance, the distance between the right eye target and left eye target is 0.269 mm. The projection amount of a stereo target from a 3D monitor is calculated by using the following equation: 0.269 : 65 = *X* : (2500 − *X*), *X* = 10.30 mm. In the case of a 5.0 m test distance, the distance between the right eye target and left eye target is 0.538 mm (0.269 × 2). The projection amount of the stereo target from the 3D monitor is calculated by using the following equation: 0.538 : 65 = *X* : (5000 − *X*), *X* = 41.04 mm. In the case of a 7.5 m test distance, the distance between the right eye target and left eye target is 0.807 mm (0.269 × 3). The projection amount of the stereo target from the 3D monitor is calculated by using the following equation: 0.807 : 65 = *X* : (7500 − *X*), *X* = 91.97 mm. The projection rates of the stereo target from the 3D monitor (projection amount of the stereo target from the 3D monitor/test distance × 100) were 0.41%, 0.82%, and 1.23%, respectively. As the test distance increased, the projection rates of the stereo target from the 3D monitor increased, and the stereo target could be recognized more easily.

Stereopsis is the most difficult binocular function parameter to evaluate. If stereoacuity can be estimated, we can be confident that other binocular functions are good. Parallax is currently used as an evaluation axis of stereoacuity. However, in different stereoacuity test conditions, parallax may be the same [35–38]. Our results show that the ease of determining stereoacuity was different under various conditions of target size and test distance. Stereoacuity should be estimated not only by parallax, but also by other elements, including test distance and target size.
